# A Rare Anatomical Variation: Intrathoracic Liver Caudate Lobe: A Case Report

**DOI:** 10.34172/aim.31604

**Published:** 2024-12-01

**Authors:** Beyza Yaşar Akpınar, Hüseyin Umut Baştürk, Şeyma Başlılar, Yahya Baraç, Bengü Şaylan

**Affiliations:** ^1^Department of Pulmonary Medicine, University of Health Sciences Sultan Abdulhamid Han Training and Research Hospital, İstanbul, Türkiye; ^2^Department of Radiology, University of Health Sciences Sultan Abdulhamid Han Training and Research Hospital, İstanbul, Türkiye; ^3^Department of Pulmonary Medicine, University of Health Sciences Süreyyapaşa Training and Research Hospital, İstanbul, Türkiye

**Keywords:** Chest pain, Computed tomography, Liver caudate lobe, Magnetic resonance imaging

## Abstract

We present a case with an intrathoracic caudate lobe in a 45-year-old woman who referred with a complaint of nonspecific chest pain. She denied previous history of trauma or surgery. Computed tomography (CT) pulmonary angiography showed a paravertebral, soft tissue mass which raised suspicion of a pleural tumor. In the coronal plane, the mass was protruding to the abdomen and had similar density with liver. Magnetic resonance imaging confirmed the diagnosis.

## Introduction

 The intrathoracic abnormally positioned liver lobe is an extremely rare anomaly which may be misdiagnosed as a mediastinal mass, pulmonary sequestration, esophageal cyst, hydatid cyst and pleural or lung tumor. The first case was described in 1957^1^ and, since then, about 30 case reports have been published.^2-4^ The most frequent symptoms are cough, respiratory distress and chest pain; but hemoptysis, dyspnea and abdominal pain have been also reported,^2^ while there are a few asymptomatic cases diagnosed incidentally^5,6^ or during autopsy.^7,8^ It may be a developmental anomaly or the liver tissue may protrude into the thoracic cavity following trauma or surgery.

 Contrast enhanced thorax computed tomography (CT), CT-angiography, and magnetic resonance imaging (MRI) are useful in making the diagnosis. However, in most cases, the diagnosis is made surgically (thoracotomy/laparotomy). We present a case with an intrathoracic accessory caudate lobe in a 45-year-old woman who referred with a nonspecific chest pain. CT showed a paravertebral, ellipsoid, well-circumscribed homogeneous soft tissue mass which raised suspicion of a pleural tumor. In the coronal plane, the mass was protruding to the abdomen and had similar density with liver. The diagnosis was confirmed with contrast-enhanced MRI of the thorax and abdomen which showed the mass to be connected with a small pedicle to the orthotopic liver.

## Case Report

**Figure 1:**
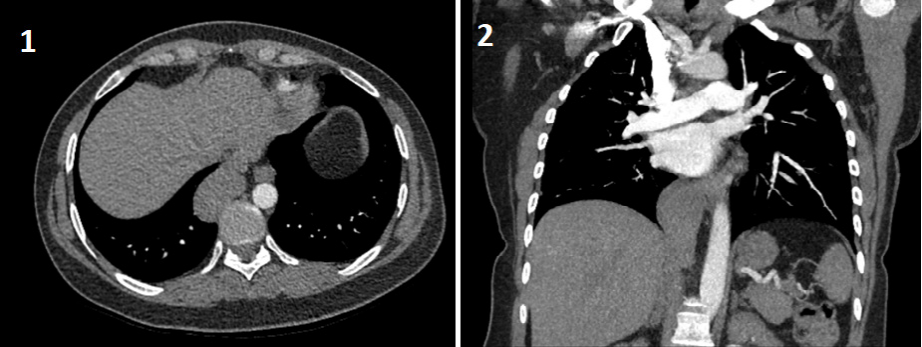
1) Non-contrast chest-CT imaging in transverse section. 2) Contrast-enhanced CT-pulmonary angiography image in coronal section

**Figure 2:**
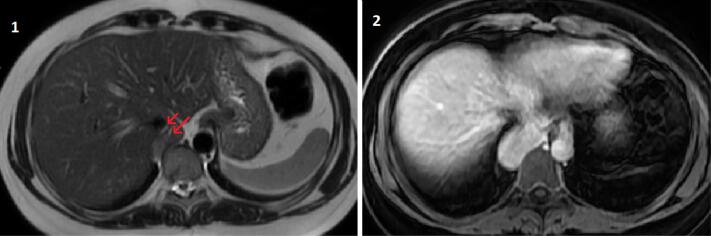
1) Vascular structures marked with red arrows, similar to liver tissue and extending into the hiatus, on the T2-weighted sequence. 2) Lesion showing similar enhancement as liver parenchyma on contrast-enhanced fat-suppressed T1-weighted sequence

 A 45-year-old female patient visited the emergency room with a right sided non-specific chest pain which she experienced when she became excited. She was an active smoker (15 pack-years) and used oral contraceptives and anxiolytics regularly. There was a family history of malignancy. CT-pulmonary angiography was requested due to the suspicion of pulmonary thromboembolism which showed no thrombus in pulmonary arteries but reported a mass lesion at the base of the right hemithorax (Figure 1). A Positron emission tomography–computed tomography (PET-CT) scan was requested which did not show any pathological fluorodeoxyglucose (FDG) uptake (SUV max: 2,1). So she was referred to our clinic. Her physical examination was unremarkable. The patient’s CT images were reevaluated; the mass was paravertebrally placed, ellipsoid, well-circumscribed and homogeneous. In the coronal plane, the mass was protruding to the abdomen and had similar density with liver (Figure 1). The patient was referred to the radiology clinic with a preliminary diagnosis of intrathoracic ectopic liver. In line with the suggestion of the radiologist, a contrast enhanced magnetic resonance imaging of the thorax and upper abdomen was performed. The mass was connected with a small pedicle to the orthotopic liver and had similar and continuous vascularity with the liver, showed similar contrast enhancement with the liver parenchyma on the contrast-enhanced fat-suppressed T1-weighted sequence, and had anatomical continuity with the caudate lobe (Figure 2). So, it was concluded that this mass was an intrathoracic liver caudate lobe.

## Discussion

 The intrathoracic displacement of a liver lobe was first reported by Hansbrough and Lipin ^1^ nearly seventy years ago but the classification of this anomaly was defined recently by Adin et al as follows: Type I: herniated liver tissue through a diaphragmatic defect; Type II: supradiaphragmatic liver mass connected to the liver through a vasculobiliary pedicle; and Type III: ectopic liver tissue separated from the liver, generally more distant. Type II has been reported as the most common, and type III the least common.^9^ Types I and II were termed as accessory liver lobe while the last type was termed as ectopic liver as it has no connection to the orthotopic liver. Our case was consistent with type II anomaly.

 The accessory liver may protrude into the thoracic cavity via diaphragmatic defects or through the inferior vena cava foramen.^2,5^ Most of the reported cases were placed in the right hemithorax. Although it was suggested that the liver tissue may be displaced following surgery or trauma, the majority of cases were suggested to be developmental.^2^ It was brought forward that in case of developmental anomalies, an accessory liver lobule was developed and the original one became atrophied.^4^ Some of the cases were reported to accompany other anomalies such as pulmonary sequestrations, arterio-venous malformations and cardiac anomalies.^2-5,8,10^

 According to the literature, most of the cases referred with complaints of chest/abdominal pain and respiratory distress. Furthermore, symptoms related to the accompanying anomalies such as productive cough and recurrent pneumonia in case of accompanying pulmonary sequestrations were also reported^2^. On the other hand, some of the cases were detected incidentally or during autopsy.^3-5,7,8^ Most of the cases reported in the literature were misdiagnosed as tumors, pulmonary sequestration or cysts; thus, the diagnosis was made via unnecessary thoracotomies or laparotomies.^2^ Adin et al proposed that the diagnosis may be made easily with all imaging techniques in case of type I and II anomalies and ultrasound examination should be preferred in the pediatric population to avoid irradiation.^9^ For radiological diagnosis, multiplanar reconstruction of the CT scans is important which may show the vascularity and the connection of the ectopic tissue to the orthotopic liver. In our case, the CT scan showed a well-circumscribed, homogeneous mass in the base of right hemithorax which had a similar density with the normal liver tissue. In coronal plane sections, the mass was connected to the caudal lobe of the liver. The MRI sequences supported our diagnosis visualizing the vascularization and connection of the mass to the liver more precisely.

 An intrathoracic ectopic or accessory liver lobe is usually clinically insignificant, so no intervention is necessary unless there is a concomitant anomaly which requires surgical treatment such as pulmonary sequestration. However, most of the cases in the literature underwent unnecessary surgeries such as thoracotomy or laparotomy.

 We presented this case to emphasize that the intrathoracic ectopic liver should be kept in mind in differential diagnosis of mass lesions adjacent to the diaphragm. The diagnosis may be achieved by noninvasive imaging techniques which may prevent unnecessary interventions and surgical complications.
